# Supplementary material for: A Prospective Observational Study of a 2-Week Integrative Inpatient Therapy on Patients with Fibromyalgia Syndrome
Source: Biomedicines. 2025 Sep 2;13(9):2144. doi: 10.3390/biomedicines13092144 (PMC12467844; doi:10.3390/biomedicines13092144)
Supplement: Supplementary file 1 [file biomedicines-13-02144-s001.zip › biomedicines-3750127-supplementary.pdf]

Supplementary table. Effect sizes

**Key outcome variable**	**Effect size** (η_p^2)<br>Main effect	**Effect size** (d_z)<br>T1-T2	**Effect size** (d_z)<br>T1-T3	**Effect size** (d_z)<br>T2-T3
Overall pain intensity (v. Korff)	.13	.62	.47	.09
Current pain intensity (v. Korff)	.34	1.23	.34	.78
Painful body sites (FSQ)	.06	.31	.17	.15
Fatigue (MFI-20)	.13	.51	.20	.27
Anxiety (HADS-A)	.21	.64	.39	.24
Depression (HADS-D)	.21	.60	.33	.25
Perceived Stress (PSS-10)	.07	.08	.30	.22
Helplessness (PSS-10)	.10	.04	.36	.32
Self-efficacy (PSS-10)	.03	.17	.20	.03
Physical QoL (SF-12)	.14	.57	.33	.19
Mental QoL (SF-12)	.11	.05	.40	.35
Current work ability	.08	.34	.27	.09
